# Dual-Domain Adaptive Input Perturbation Sensitivity for Adversarial Example Detection

**DOI:** 10.3390/s26144467

**Published:** 2026-07-14

**Authors:** Li Yue, He Gao, Hao Wang, Ming Yang, Dawei Xu

**Affiliations:** College of Computer Science and Technology, Changchun University, Changchun 130022, China

**Keywords:** adversarial example detection, deep neural networks, input perturbation sensitivity, manifold-motivated micro-scale probing, dual-state sensitivity fusion, temperature scaling

## Abstract

Vision-sensor-based intelligent perception systems are increasingly used in safety-critical scenarios such as autonomous driving, edge surveillance, and Internet-of-Things (IoT) platforms. The vulnerability of deep neural networks to adversarial examples raises security concerns for sensor-acquired visual data in such systems, motivating the study of output-probability-based adversarial example detection methods under controlled benchmark settings. Existing input-level sensitivity detection methods generally rely on static perturbation scales or single-state metrics. When confronted with heterogeneous attacks, such as one-step attacks and iterative attacks, as well as complex tasks with high class density, these methods often suffer from unstable metric directions and insufficient boundary probing capability. To address these issues, this paper proposes a dual-domain adaptive adversarial example detection method based on Multi-scale Input Sensitivity (MSIS). The proposed method introduces a Manifold-Motivated Micro-scale Probing (MMP) mechanism and a Dual-State Sensitivity Fusion (DSF) mechanism. MMP adopts a task-level perturbation scaling strategy motivated by the compressed inter-class manifold structures observed in high-density classification tasks, thereby alleviating perturbation overflow and improving boundary probing effectiveness. DSF employs temperature scaling to extract sensitivity features under both the native state and the smoothed state, and alleviates the directional conflict of heterogeneous attacks under a single metric through dual-state joint modeling. Experimental results demonstrate that, without modifying the parameters of the target model, the proposed method achieves favorable detection performance against representative attacks, including FGSM, PGD, and C&W, on the CIFAR-10 and CIFAR-100 datasets. Taking the CIFAR-10 + ResNet-18 configuration as an example, the detection AUC of the proposed method against the PGD attack reaches 97.75%, an improvement of 24.32 percentage points over the best-performing non-intrusive baseline method, Energy Score (73.43%), with the lowest FPR@95TPR dropping to 7.75%. Under the CIFAR-10 + ResNet-50 configuration, the detection AUC against the PGD attack further reaches 99.14%. Meanwhile, even when compared with PASA (2024), the latest intrusive method requiring access to model gradients, the average AUC of the proposed method on CIFAR-10 + ResNet-18 (97.49%) is still 18.81 percentage points higher, and its inference latency is only 1/11th that of PASA. These results suggest that introducing task-level spatial-domain scaling and temperature-state adaptation can improve output-probability-based adversarial example detection under non-intrusive benchmark settings, providing algorithmic evidence for output-probability-based detection of adversarial perturbations in visual classification tasks.

## 1. Introduction

Vision-sensor-based intelligent perception systems are widely adopted in safety-critical scenarios such as autonomous driving, smart surveillance, and edge Internet-of-Things (IoT) platforms [1], making the security of sensor-acquired visual data an increasingly important research concern. Benefiting from the powerful representation capability of deep neural networks (DNNs), modern visual sensing systems can achieve highly accurate perception and decision support. However, recent studies have shown that DNNs are highly vulnerable to adversarial examples (AEs), where carefully crafted perturbations can induce classifiers to produce incorrect predictions with high confidence while remaining almost imperceptible to humans [2]. Such vulnerabilities motivate the study of adversarial example detection methods for sensing-related visual classification tasks, especially under non-intrusive settings where only output probabilities of the target model are available. Among existing defense strategies, detection-based defenses have become an important research direction due to their relatively low deployment cost and the fact that they do not require modification of the internal parameters of the target model. In parallel, broader robustness research has also investigated certified and formally verifiable defenses, which provide complementary perspectives on the reliability of neural networks under adversarial perturbations [3]. Representative input-perturbation-based detection methods, such as Feature Squeezing [4], exploit the differences in local smoothness between natural images and adversarial examples, and perform lightweight detection by observing prediction consistency after preprocessing operations.

However, existing input-level sensitivity detection methods generally rely on static probing assumptions and exhibit two major blind spots in practical defense scenarios. The first is the spatial-domain blind spot. In high class-density tasks, such as CIFAR-100, the decision manifold becomes highly crowded, and fixed perturbation scales can easily cross dense decision boundaries, resulting in boundary probing failure. The second is the temperature-state blind spot. Different adversarial attacks exhibit significantly different geometric characteristics. One-step attacks, such as FGSM [5], and strong iterative attacks, such as PGD [6], often demonstrate opposite sensitivity trends in a static feature space. The conflict introduced by such heterogeneous characteristics can cause traditional one-directional threshold-based detection to suffer from the problem of metric inversion, thereby limiting the generalization capability of detectors against unknown attacks.

To address these limitations, this paper proposes an adversarial example detection framework based on Multi-scale Input Sensitivity (MSIS). The proposed framework combines task-level spatial-domain probing with temperature-state feature modeling to characterize output-probability responses under heterogeneous attacks. To address the spatial-domain blind spot, a Manifold-Motivated Micro-scale Probing (MMP) mechanism is designed to perform task-level scaling of the perturbation probing set, motivated by the observation that high-density tasks exhibit compressed inter-class manifold structures, thereby reducing perturbation overflow in dense decision regions. To address the temperature-state blind spot, a Dual-State Sensitivity Fusion (DSF) mechanism is proposed. DSF employs temperature scaling [7] to extract sensitivity features under the native state (T=1) and the smoothed state (T=5), respectively. By constructing a linear discriminative space and jointly modeling dual-state features, the proposed mechanism alleviates the metric conflict caused by heterogeneous attacks at the feature-space level.

The main contributions of this paper are summarized as follows:

- The metric inversion phenomenon in existing input-level detection methods under heterogeneous attacks is analyzed, together with the limitations of spatial boundary probing in high class-density tasks.
- A Manifold-Motivated Micro-scale Probing (MMP) mechanism is proposed. Through task-level spatial-domain scaling motivated by inter-class manifold density, MMP reduces perturbation overflow in high-density classification tasks and provides a more suitable probing scale for sensitivity-based detection.
- A Dual-State Sensitivity Fusion (DSF) mechanism is proposed. By jointly modeling sensitivity features under the native and temperature-smoothed states, DSF mitigates metric-direction conflicts observed under heterogeneous attacks.
- Experimental results demonstrate that, without modifying the parameters of the target model, the proposed MSIS framework achieves effective detection performance against representative attacks, including FGSM [5], PGD [6], and C&W [8], on the CIFAR-10 and CIFAR-100 benchmark datasets. These results provide controlled benchmark evidence for output-probability-based adversarial detection under heterogeneous attack settings.

## 2. Related Work

### 2.1. Adversarial Detection Based on Input Transformation

Input-transformation-based detection mechanisms identify anomalous samples by applying specific reconstructions to input data and measuring the discrepancy between model predictions before and after transformation. Owing to their plug-and-play nature and the fact that they do not require modification of the internal parameters of the target model, such methods have become an important direction in lightweight adversarial defense. A representative example is Feature Squeezing [4], which compresses the input feature space through bit-depth reduction and spatial smoothing operations to suppress the fine-grained local structures of adversarial perturbations. More recent purification-based defenses further exploit generative reconstruction mechanisms, such as diffusion-model-based adversarial purification [9], to remove adversarial perturbations before classification. However, these methods mainly rely on empirically selected static transformation parameters and a single decision threshold. When confronted with heterogeneous attacks, they often struggle to balance perturbation suppression and preservation of natural features, thereby limiting detection generalization capability.

Beyond explicit input transformation, another category of input-level detection methods injects random perturbations into input samples and characterizes local sample stability by measuring variations in classifier outputs under perturbations [10,11,12]. Other recent adversarial image detection studies have also explored reconstruction-based black-box detection [13] and semantic-response-based detection cues [14], indicating the increasing diversity of non-standard detection signals. Compared with conventional preprocessing-based approaches, such sensitivity-analysis-based methods can capture more continuous boundary response information. Nevertheless, they remain fundamentally constrained by static probing assumptions. Specifically, their probing scales are usually fixed in advance, which can easily cause boundary probing failure due to perturbation overflow in high class-density tasks. In addition, these methods rely on sensitivity statistics under a single state. When facing heterogeneous attacks with significantly different geometric distributions, one-directional metrics are prone to suffering from metric inversion. Therefore, establishing a dynamic detection mechanism that jointly considers task-level spatial-domain scaling and temperature-state feature alignment has become critical for overcoming the limitations of existing input-level detection methods.

### 2.2. Heterogeneity and Geometric Characteristics of Adversarial Attacks

Due to differences in optimization strategies, perturbation generation mechanisms, and attack objectives, adversarial attacks often exhibit significant geometric heterogeneity in feature space. Such heterogeneity not only affects the distribution patterns of adversarial samples but also directly determines their prediction response behavior under random perturbations. Understanding these response differences among attacks is fundamental for analyzing the limitations of existing static detection methods.

Typical one-step attacks, such as FGSM [5], generate adversarial examples by applying a single perturbation along the gradient direction of the loss function. Due to the lack of iterative optimization, such attacks are generally unable to precisely align with complex decision boundary structures. Empirically, these samples often exhibit relatively weak prediction fluctuations under random perturbations, and their sensitivity responses may even be lower than those of certain natural samples. In contrast, strong iterative attacks, such as PGD [6], progressively approach target class regions through multi-step projected optimization. Their outputs are usually associated with high confidence and may exhibit response patterns substantially different from those of one-step attacks during perturbation probing.

Furthermore, optimization-based boundary attacks, such as C&W [8], aim to minimize perturbation magnitude. The generated adversarial examples are therefore typically located closer to class decision boundaries and possess relatively small local decision margins, making them more sensitive to random perturbations. These observations indicate that one-step attacks, iterative attacks, and boundary attacks do not exhibit a unified perturbation response trend, but instead display substantial heterogeneity.

Such response heterogeneity poses a major challenge to existing static probing mechanisms. Under a single-state sensitivity metric framework, different attacks may produce response patterns with opposite directions. For example, boundary attacks often exhibit pronounced degradation in prediction consistency, whereas certain one-step attacks may demonstrate relatively weak degradation trends. When detectors continue to rely on one-directional static thresholds, such inconsistencies in response direction can easily lead to metric inversion, thereby undermining the stability of a unified detection criterion.

The above analysis suggests that static sensitivity metrics under the native state alone are insufficient for simultaneously handling heterogeneous attack patterns. To achieve unified representation of different attack responses, it is necessary to introduce a feature decoupling mechanism in the state space and alleviate metric conflicts caused by heterogeneous attacks through multi-state joint feature modeling. This observation also provides the theoretical motivation for the subsequent Dual-State Sensitivity Fusion mechanism.

### 2.3. Decision Manifolds and Temperature Scaling

The classification process of deep neural networks can generally be viewed as a manifold partitioning problem in high-dimensional feature space. As task complexity increases, particularly in high class-density scenarios, inter-class decision regions in feature space tend to become compressed, resulting in significantly reduced local decision margins. Under such conditions, the noise introduced by traditional fixed-scale perturbation probing can easily exceed the locally stable region of a sample, causing additional class transitions and weakening the detector’s ability to characterize genuine boundary vulnerability. Existing studies have shown that the local geometric structure of decision manifolds is strongly coupled with perturbation probing scales [15]. When the probing scale is mismatched with the local margin, input perturbations may not effectively characterize boundary sensitivity and may instead introduce additional probing bias.

Motivated by this observation, recent robustness analysis studies have increasingly focused on the influence of decision manifold geometry, suggesting that the effectiveness of perturbation probing largely depends on its compatibility with local boundary structures. In particular, in high class-density tasks, the reduction of decision boundary spacing further amplifies the limitations of fixed-scale probing. This consensus provides theoretical support for adjusting perturbation scales according to task-level manifold density and demonstrates that input sensitivity analysis should move beyond fixed global probing assumptions toward task-level spatial-domain probing mechanisms compatible with dense decision manifolds.

In addition to decision manifold geometry, the calibration state of model output confidence also substantially influences the quality of detection feature representations. Temperature Scaling [7] was originally introduced for uncertainty calibration in neural networks. By incorporating a temperature parameter into the Softmax mapping, it smooths the output distribution and alleviates the overconfidence problem of deep models. Subsequent studies further demonstrated that the temperature parameter not only affects distribution calibration but also changes the dynamic range of output responses [16]. In particular, for anomalous features generated through nonlinear optimization, temperature adjustment can effectively alter local response gradients. Consequently, the temperature state can serve not only as a calibration tool but also as a potential mechanism for revealing adversarial characteristics.

Following this line of research, several studies have explored extending temperature calibration beyond uncertainty calibration to broader feature representation analysis in order to enhance the observability of hidden response patterns. By obtaining multidimensional output responses under different temperature states and jointly exploiting features from multiple states, prior work has shown that the limitations of single-state representations can be effectively alleviated [16]. This direction directly provides theoretical support for temperature-state-domain feature modeling and offers a potential solution to the metric conflicts introduced by heterogeneous attacks.

In summary, the density characteristics of decision manifolds demonstrate the necessity of adjusting perturbation scales in accordance with task-level boundary density, while temperature scaling reveals the potential of multi-state output responses for enhancing feature representation capability. Together, these two perspectives establish the theoretical foundation for the dual-domain adaptive detection mechanism proposed in this paper.

## 3. Multi-Scale Input Sensitivity (MSIS) Detection Framework

To overcome the blind spots of existing static probing methods in both the spatial domain and the temperature-state domain, this paper proposes a Multi-scale Input Sensitivity (MSIS) detection framework equipped with a dual-domain adaptive mechanism. This section introduces the overall architecture and the fundamental sensitivity metric of the framework, followed by two core components: Manifold-Motivated Micro-scale Probing (MMP) and Dual-State Sensitivity Fusion (DSF).

### 3.1. Overall MSIS Framework and Fundamental Sensitivity Metric

**Figure 1:**
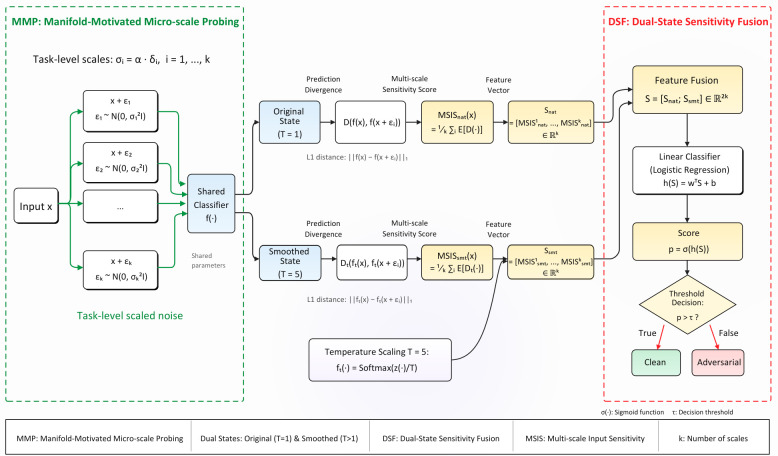
**Multi-scale Input Sensitivity (MSIS) Detection Framework.** The diagram illustrates the complete pipeline from task-level MMP scaling and dual-state sensitivity extraction to logistic-regression-based adversarial detection.

As illustrated in Figure 1, the proposed Multi-scale Input Sensitivity (MSIS) detection framework distinguishes adversarial manifolds from natural manifolds by actively injecting random perturbations into the input space and characterizing multi-scale response variations in the output probability space. The overall detection process consists of three major stages. First, the micro-scale perturbation range is determined at the task level based on classification density of the target task. Second, multi-scale random perturbations are applied to input samples, and sensitivity features of prediction responses are extracted under different temperature states. Finally, adaptive discrimination is performed in the joint feature space.

Essentially, the sensitivity metric characterizes the response variation induced in the output probability space by minor perturbations in the input space. For a given input sample *x*, let the logits of the classifier be denoted as z(x). The corresponding probability output is defined as:

(1) p(x)=Softmax(z(x))

Random perturbations following a zero-mean Gaussian distribution are injected into the input space:

(2) $$\epsilon ∼N(0,\sigma^{2}I)$$

where σ is the scale parameter controlling the perturbation intensity. Under a given perturbation scale σ, the corresponding local sensitivity is defined as:

(3) $$S\left(x,\sigma \right)=E_{\epsilon ∼N(0,\sigma^{2}I)}\left[{∥p\left(x\right)-p\left(x+\epsilon \right)∥}_{1}\right]$$

In practice, the above expectation is approximated using *K* Monte Carlo samples:

(4) $$S\left(x,\sigma \right)\approx \frac{1}{K}\sum_{k=1}^{K}{∥p\left(x\right)-p\left(x+\epsilon_{k}\right)∥}_{1},\;\epsilon_{k}∼N\left(0,\sigma^{2}I\right)$$

Furthermore, a probing set consisting of *N* increasing perturbation scales is constructed as:

(5) $$\Sigma =\{\sigma_{1},\sigma_{2},\ldots ,\sigma_{N}\}$$

Thus, the sensitivity representation of the sample under multi-scale perturbations is obtained as:

(6) $$S\left(x\right)=\left[S\left(x,\sigma_{1}\right),S\left(x,\sigma_{2}\right),\ldots ,S\left(x,\sigma_{N}\right)\right]\in R^{N}$$

The resulting multi-scale feature vector characterizes the stability structure of a sample under different perturbation intensities and effectively reflects its local geometric properties on the decision manifold. It also serves as the fundamental representation for subsequent task-level spatial-domain scaling (MMP) and temperature-state-domain feature fusion (DSF).

### 3.2. Manifold-Motivated Micro-Scale Probing Mechanism (MMP)

Existing methods generally adopt a globally fixed probing set Σ [4,10]. However, in high class-density tasks such as CIFAR-100, the distances between class centers become significantly compressed, resulting in compressed inter-class decision regions. When fixed perturbations are excessively large for such dense regions, even natural samples may cross decision boundaries, leading to probing failure.

To address this issue, this paper proposes a Manifold-Motivated Micro-scale Probing (MMP) mechanism. Motivated by the observation that high-density classification tasks exhibit compressed inter-class manifold structures, MMP adopts a task-level perturbation scaling strategy to alleviate perturbation overflow in dense classification settings. Instead of performing instance-level margin estimation, MMP determines a fixed scaling coefficient α through a one-time validation-set grid search, which remains constant during inference. Specifically, MMP performs task-level down-scaling on the original probing set to construct a micro-scale probing set:

(7) $$\Sigma_{micro}=\left\{\alpha \sigma_{1},\alpha \sigma_{2},\ldots ,\alpha \sigma_{N}\right\}$$

where α∈(0,1) denotes a task-level scaling coefficient. In practice, α is determined through an offline grid search on a validation set, with the candidate set defined as α∈{0.01,0.02,0.05,0.1,0.2}. The optimal value is selected according to the lowest FPR@95TPR on the validation set. Once selected for a given dataset setting, α remains fixed for all test samples and is not re-tuned for individual samples. The optimal α is closely related to task class density. In low-density tasks such as CIFAR-10, inter-class distances are relatively large, and excessively small α values are insufficient to induce distinguishable local sensitivity responses from adversarial examples. Experimental results show that α=0.05 achieves the best balance between activating sensitivity responses and preserving the distribution of clean samples. In contrast, for high-density tasks such as CIFAR-100, decision boundaries become highly crowded. When α>0.02, the prediction outputs of clean samples tend to drift toward neighboring classes due to excessive perturbation noise, leading to uncontrollable false positive rates. Consequently, a more conservative setting of α=0.02 is adopted. The above search procedure is performed only once on the validation set and does not involve any modification of the target model parameters.

When α=1, the proposed mechanism degenerates into the standard multi-scale probing formulation. Through this task-level scaling, MMP helps the injected perturbations probe metastable adversarial boundaries while reducing perturbation overflow in dense decision regions. As a result, the proposed mechanism alleviates probing overflow in dense manifolds and improves boundary perception capability in complex tasks.

It should be noted that MMP operates at the task level rather than the instance level: the scaling coefficient α is determined once via an offline validation-set grid search and remains fixed during inference. The held-out evaluation split is not used for selecting α. This design avoids gradient access or iterative boundary search, thereby preserving the non-intrusive nature of the detector. Per-sample margin estimation may further improve local adaptivity, but it would introduce additional computational cost and is left for future work.

### 3.3. Dual-State Sensitivity Fusion Mechanism (DSF)

After addressing the blind spot in the spatial domain, it is still necessary to resolve the response conflicts caused by heterogeneous attacks in feature space. Due to the anomalous perturbation stability exhibited by one-step attacks and the artificially high confidence associated with strong iterative attacks, sensitivity metrics under a single state are highly susceptible to directional inversion. The proposed DSF mechanism addresses this issue through feature decoupling and joint modeling in the temperature-state domain.

#### 3.3.1. Temperature Scaling and Dual-State Feature Extraction

Given the logit output z(x) before the classification layer, a temperature parameter *T* is introduced to recalibrate the Softmax probability distribution as follows [7]:

(8) $$f_{T}{\left(x\right)}_{i}=\frac{\exp(z{\left(x\right)}_{i}/T)}{\sum_{j=1}^{C}\exp\left(z{\left(x\right)}_{j}/T\right)}$$

The DSF mechanism extracts sensitivity features under two representative states:

- **Native state** (T=1): The original decision confidence is preserved to sensitively capture the subtle degradation caused by boundary attacks under extremely small margins. The extracted feature is denoted as $v_{\mathrm{native}}$.
- **Smoothed state** (T=5): The output distribution is flattened through a higher temperature, which suppresses the artificially high confidence of strong iterative attacks and exposes their underlying nonlinear response gradients. The extracted feature is denoted as $v_{\mathrm{smooth}}$.

#### 3.3.2. Joint Feature Discrimination and Direction-Adaptive Correction

The multi-scale sensitivity scores extracted under the above two states are concatenated to construct a dual-state feature vector $V=[v_{native},v_{smooth}]$. Subsequently, a linear logistic regression classifier is introduced to perform joint discrimination in the extended dual-state feature space:

(9) $$P\left(\mathrm{adversarial}|x\right)=\mathrm{Sigmoid}\left(W^{T}V+b\right)$$

By searching for an optimal separating hyperplane in the joint feature space, the linear model can adaptively assign reverse penalties to features exhibiting abnormal responses according to their state-dependent contributions. When certain one-step attacks exhibit anomalously low sensitivity, the model can automatically correct the metric direction by learning corresponding negative weights.

Finally, the detection decision is obtained by comparing the joint classification probability with a threshold γ:

(10) $$\hat{y}=\left\{\begin{array}{cc} 1, & P(\mathrm{adversarial}|x)>\gamma \\ 0, & \mathrm{otherwise} \end{array}\right.$$

where label 1 denotes adversarial examples and label 0 denotes normal samples. The threshold γ is determined on the validation split when a binary operating point is required. For threshold-independent AUC evaluation, γ is not used. Through this joint discrimination mechanism, the metric inversion phenomenon under a single state can be automatically corrected at the feature-space level, thereby enabling unified discrimination against heterogeneous attacks.

## 4. Experimental Design and Results Analysis

### 4.1. Experimental Setup and Evaluation Benchmarks

#### 4.1.1. Datasets and Target Models

To comprehensively evaluate the detection performance of the proposed method under different task complexities and model capacities, experiments are conducted on two representative image classification datasets: CIFAR-10 and CIFAR-100 [17]. CIFAR-10 contains natural images from 10 categories, whereas CIFAR-100 extends to 100 fine-grained categories with substantially increased class density, thereby providing a suitable benchmark for evaluating detection performance under high-density decision manifold scenarios.

Although standardized robustness benchmarks such as RobustBench [18] provide unified evaluation protocols for adversarial robustness, this paper focuses on output-probability-based adversarial example detection under controlled CIFAR benchmark settings.

For model configuration, two representative convolutional neural networks are selected as the main target classifiers: ResNet-18 and ResNet-50 [19]. The former adopts a relatively lightweight architecture with limited representation capacity, while the latter employs a deeper residual structure with stronger expressive capability. Evaluation across models with different capacities enables assessment of the generalization capability of the proposed detection framework under diverse residual-network architectures. In addition, to partially examine whether the proposed detector is tied to residual architectures, we further include an additional VGG-16-BN experiment on CIFAR-10. To further provide a preliminary check beyond convolutional architectures, we also evaluate MSIS on a lightweight Vision Transformer (ViT) backbone trained on CIFAR-10. It should be noted that the VGG-16-BN and lightweight ViT experiments are intended as limited architectural stress tests, rather than substitutes for ImageNet-scale validation or comprehensive evaluation over large transformer backbones. All models are independently trained on the corresponding datasets using standard training procedures and achieve stable classification performance on clean test sets.

For the scaling coefficient in MMP, validation-set grid search determines α=0.05 for CIFAR-10 and α=0.02 for CIFAR-100. These values are selected once at the dataset level and then fixed across all target models, attack types, and held-out evaluation folds within the corresponding dataset.

All detection experiments in this paper are conducted on fixed pre-trained classification models, and the entire detection process does not require any modification of model parameters.

#### 4.1.2. Attack Configuration and Adversarial Sample Quality Evaluation

To construct representative adversarial sample sets, three typical attack methods are selected, corresponding to different optimization depths and geometric characteristics:

- **FGSM (one-step attack)** [5]: Adversarial examples are generated through a single gradient-sign perturbation step. This method has low computational cost but limited capability in precisely aligning with decision boundaries.
- **PGD (iterative attack)** [6]: Multi-step projected iterative optimization is employed to approximate optimal attacks within the $L_{\infty }$-constrained space. Its attack strength is substantially higher than that of one-step methods.
- **C&W (optimization-based boundary attack)** [8]: By minimizing perturbation magnitude, adversarial examples are generated near decision boundaries with high stealthiness, posing significant challenges to detection methods.

To ensure the validity of experimental evaluation, the quality of generated adversarial samples is uniformly constrained and verified. Specifically, all attacks are performed under standard perturbation norm constraints, and sample effectiveness is evaluated using the Attack Success Rate (ASR). Only adversarial examples that successfully induce misclassification are retained for subsequent detection experiments, thereby preventing weak attacks or ineffective perturbations from interfering with detection results. Detailed attack parameter settings and sample statistics are provided in Table 1.

#### 4.1.3. Baseline Methods and Evaluation Metrics

To comprehensively evaluate the performance of the proposed method, several representative lightweight adversarial detection methods are selected as baseline approaches, covering different types of discrimination mechanisms:

- **Feature Squeezing** [4]: A detection method based on input transformation and spatial compression.
- **Maximum Softmax Probability (MSP)** [20]: A detection method based on the maximum confidence probability of model outputs.
- **Energy Score** [21]: A detection method based on the log-likelihood energy distribution function.
- **DBA-style Reconstruction** [13]: A recent black-box reconstruction-based detector that uses pixel-frequency reconstruction error as the anomaly score.

These methods perform sample discrimination from different perspectives, including input-space compression, confidence response, energy distribution, and reconstruction error, thereby reflecting the performance of existing lightweight and black-box detection mechanisms from multiple aspects.

For evaluation metrics, the following two standard metrics are primarily adopted to quantitatively assess detection performance:

- **AUC (Area Under the ROC Curve)**: Measures the overall discrimination capability of the detector under different thresholds. Values closer to 1 indicate better performance.
- **FPR@95TPR**: Denotes the False Positive Rate when the True Positive Rate reaches 95%, and is used to evaluate detector reliability under high-recall conditions.

It should be particularly noted that, since the proposed method ultimately produces multidimensional feature representations, a strict 5-Fold Cross-Validation strategy is adopted when computing evaluation metrics in order to ensure fairness and rigor in performance assessment. This setting completely avoids artificially inflated performance caused by classifier overfitting to known samples.

For single-score baseline detectors, the anomaly-score direction may vary across attack types due to the metric inversion phenomenon. To ensure a fair comparison, the score direction is determined only from the training split within each cross-validation fold. Specifically, if the training split indicates that lower scores correspond to adversarial samples, the score is negated before evaluation on the held-out fold. The held-out fold labels are not used to determine the score direction, thereby avoiding post-hoc correction on the evaluation data. This direction-selection rule is applied uniformly to all single-score baseline methods. For MSIS, no manual 1−AUC correction is applied, since the logistic-regression classifier learns the discriminative direction from the training split within each fold.

### 4.2. Implementation Details and Reproducibility

For reproducibility, we provide the detailed implementation protocol used in all experiments. For adversarial sample generation, FGSM is configured with ϵ=8/255, PGD is configured with ϵ=8/255, step size 2/255, and 10 iterations, and C&W is configured with c=1, learning rate 0.01, and 100 optimization steps. All adversarial samples are generated on the corresponding target classifier.

For MSIS feature extraction, Gaussian perturbations are sampled from $N(0,\sigma^{2}I)$. The base probing set contains four scales, i.e., N=4. After task-level MMP scaling, the actual probing scales are set to {0.01,0.02,0.05,0.08} for CIFAR-10 and {0.004,0.008,0.02,0.032} for CIFAR-100. For each scale, the Monte Carlo sampling number is set to K=10. The native and smoothed temperature states are fixed to $T_{1}=1$ and $T_{2}=5$, respectively.

All hyperparameters used for feature extraction are fixed before held-out evaluation. Specifically, α is selected by validation-set grid search at the dataset level, while $T_{2}=5$ is adopted according to the temperature ablation and full-MSIS temperature stability analyses. These hyperparameters are not re-selected for individual attack types or test samples.

The sensitivity feature at each scale is computed as the average $L_{1}$ distance between the output probability vector of the original input and that of its Gaussian-perturbed variants over *K* Monte Carlo samples. The full MSIS feature vector consists of one temperature-state discrepancy feature and eight multi-scale sensitivity features extracted under $T_{1}$ and $T_{2}$, resulting in a 9-dimensional feature vector. The default MSIS configuration uses the $L_{1}$ distance with Gaussian perturbations. To justify this design choice, we further compare alternative distance metrics and perturbation distributions in the ablation study, including $L_{2}$ distance, KL divergence, uniform noise, and Laplace noise. For KL divergence, the direction is fixed as KL(p(x)∥p(x+ϵ)) throughout the experiment.

For detector training and evaluation, we use stratified 5-fold cross-validation with shuffling and random seed 42. In each fold, feature standardization is performed using StandardScaler fitted only on the training split and then applied to the validation split. A logistic regression classifier is used as the default fusion classifier, with balanced class weights, maximum iteration number 2000, and random seed 42. This choice is motivated by its low computational cost, interpretability, and suitability for the compact 9-dimensional MSIS feature vector. To further examine whether more expressive nonlinear fusion models provide additional benefits, we also conduct a supplementary fusion-classifier comparison in the ablation study. Detection performance is reported using AUC and FPR@95TPR.

To further assess the stability of the proposed method with respect to random factors, we additionally conduct a repeated-run analysis on the CIFAR-10 + ResNet-18 configuration using five random seeds, i.e., seeds 0–4. In this analysis, the random seed controls Monte Carlo perturbation sampling, cross-validation splitting, and logistic-regression initialization. The mean and standard deviation of AUC and FPR@95TPR are reported to quantify the sensitivity of MSIS to random evaluation procedures.

To reduce randomness in performance estimation, all reported detector results are obtained using the same stratified cross-validation protocol and fixed random seed. The Monte Carlo perturbation sampling number is fixed to K=10 for all scales and datasets. Since the same data splits, feature standardization procedure, and classifier configuration are applied consistently across methods, the reported comparisons reflect relative performance under a controlled evaluation protocol.

For single-score baselines, score-direction selection is also performed within each training fold and then applied to the corresponding validation fold, ensuring that no label information from the held-out evaluation split is used for direction correction.

The same MSIS feature-extraction and evaluation protocol is used for both the main comparison experiments and the ablation studies, ensuring that the reported full-model results are consistent across tables.

### 4.3. Experimental Results and Analysis

To validate the effectiveness of the proposed framework, extensive comparative experiments are conducted on the CIFAR-10 and CIFAR-100 datasets [17] under different target classifiers (ResNet-18 and ResNet-50) [19] and multiple attack algorithms. In addition to the original lightweight baseline methods, a recent DBA-style reconstruction-based black-box detector [13], Mahalanobis Distance [22], and PASA [23] are further introduced, enabling broader comparison across reconstruction-based, output-probability-based, feature-based, and gradient-based detection paradigms.

The AUC evaluation results of different detection methods are presented in Table 2, while the corresponding model access requirements of each method are summarized in Table 3.

To improve evaluation stability, all AUC and FPR@95TPR values are computed under the same stratified 5-fold cross-validation protocol. For each fold, detector training, score-direction selection, feature standardization, and threshold-related evaluation are performed using only the training split, and the resulting model or score direction is then evaluated on the held-out split. This protocol reduces the influence of random data partitioning and avoids using held-out labels for model selection or post-hoc score correction.

To further verify that the reported performance is not caused by a particular random split or Monte Carlo sampling instance, we repeat the MSIS evaluation on CIFAR-10 + ResNet-18 with five random seeds. The results are summarized in Table 4.

As shown in Table 4, the standard deviation of AUC across five random seeds remains below 0.02 percentage points for all three attacks, indicating that the proposed MSIS detector is highly stable with respect to Monte Carlo sampling, cross-validation partitioning, and classifier initialization. This robustness is consistent with the design of MSIS, which relies on averaging over multiple Monte Carlo samples (K=10) and multiple probing scales, reducing the influence of any single random draw on the final detection decision.

#### 4.3.1. Overall Detection Performance and Generalization Capability Evaluation

In most evaluation scenarios, the proposed method demonstrates favorable overall detection performance and stable behavior across the evaluated settings. Taking the ResNet-18 model on the CIFAR-10 dataset as an example, the proposed method achieves an AUC of 97.75% against the PGD attack [6], outperforming the best-performing non-intrusive baseline method, Energy Score (73.43%) [21], by 24.32%. The added DBA-style reconstruction baseline exhibits highly configuration-dependent behavior. It achieves strong AUC values against FGSM and PGD under the CIFAR-10 + ResNet-18 setting, but degrades substantially under the C&W attack and becomes close to random under the CIFAR-100 configurations. This suggests that reconstruction-based black-box detection can be effective for certain attack patterns, but may be sensitive to dataset complexity, model architecture, and attack type. In contrast, MSIS maintains more balanced detection performance across heterogeneous attacks and evaluation configurations.

Notably, the introduction of two intrusive methods, Mahalanobis Distance and PASA, substantially expands the comparison scope. Mahalanobis Distance [22] requires extraction of intermediate-layer features from the target model and therefore represents a typical intrusive detection method. Under the CIFAR-10 + ResNet-18 configuration, its average AUC reaches only 53.25%, which is close to random-level performance. This result suggests that Mahalanobis-distance-based metrics relying solely on intermediate-layer representations do not exhibit stable advantages in adversarial detection tasks, and their effectiveness is sensitive to model architecture and training settings.

PASA, a recent intrusive method proposed in 2024 [23], performs detection by jointly modeling prediction sensitivity and Integrated Gradients attribution sensitivity. Under the CIFAR-10 + ResNet-18 configuration, PASA achieves an average AUC of 78.68%, outperforming several contemporary non-intrusive methods, yet still remaining below the proposed method (97.49%).

These results indicate that, even when compared with intrusive methods requiring access to internal gradients or intermediate-layer representations, the proposed non-intrusive framework, which relies solely on output probability distributions, can still maintain competitive or superior detection performance in the evaluated benchmark settings. This comparison directly validates the feasibility and effectiveness of the non-intrusive deployment paradigm in practical adversarial detection scenarios.

As dataset class density increases (switching to CIFAR-100) and model depth becomes larger (switching to ResNet-50), the nonlinearity of the decision manifold increases substantially, leading to varying degrees of performance degradation for baseline methods. For example, even after directional correction for metric inversion, the average AUC of Energy Score decreases from 88.39% to 81.64% on ResNet-50, while PASA further declines to 62.76% under the same setting. In contrast, the proposed method still maintains an average AUC of 94.17% on CIFAR-100 + ResNet-50, outperforming all compared baseline methods.

It should be noted that, under the CIFAR-100 + ResNet-18 configuration, the proposed method achieves an average AUC of 82.60%, outperforming MSP (74.46%) but still showing a clear performance gap compared with the CIFAR-10 settings. This indicates that high class density and limited model capacity jointly make adversarial detection more challenging in this scenario. Compared with CIFAR-10, CIFAR-100 contains substantially finer-grained categories, which increases inter-class overlap and compresses decision regions in the learned representation space. When combined with the relatively limited representation capacity of ResNet-18, both clean and adversarial samples may exhibit less separable output-probability responses under random perturbations. Therefore, although the complete MSIS pipeline improves over single-score baselines in this setting, the remaining performance gap indicates that high-density low-capacity configurations remain challenging for output-probability-based adversarial detection. This phenomenon is related to the relatively limited representation capability of the model and the increased complexity of the decision manifold under this configuration. Notably, all methods, including intrusive approaches, exhibit relatively low overall performance in this scenario, indicating that the detection task itself is inherently challenging rather than reflecting a specific weakness of the proposed method.

To further examine whether MSIS is restricted to residual architectures, an additional architectural generalization experiment is conducted on CIFAR-10 using VGG-16-BN, a non-residual convolutional architecture. The trained VGG-16-BN model achieves 92.93% clean test accuracy.

As shown in Table 5, MSIS achieves an average AUC of 0.8713 across FGSM, PGD, and C&W attacks, outperforming MSP and Energy Score by 5.59 and 4.00 percentage points, respectively. These results suggest that the proposed output-probability sensitivity features remain applicable beyond ResNet-based architectures. Nevertheless, this experiment should be interpreted as an additional architectural generalization check rather than a comprehensive benchmark over all modern backbones; broader evaluation on Vision Transformers and ConvNeXt remains an important direction for future work.

To further examine whether MSIS can be applied beyond convolutional architectures, we conduct an additional preliminary experiment using a lightweight Vision Transformer backbone on CIFAR-10. The model is trained from scratch on CIFAR-10 and achieves a clean test accuracy of 78.99%. This experiment is intended as a small-scale architectural stress test rather than a full ImageNet-scale transformer evaluation.

As shown in Table 6, MSIS achieves the highest average AUC of 81.94% under the lightweight ViT setting, outperforming MSP and Energy Score by 12.18 and 20.72 percentage points, respectively. This result suggests that the proposed output-probability-based sensitivity features can still provide useful detection information under a transformer-style backbone. However, the results should be interpreted conservatively. First, the lightweight ViT is trained from scratch on CIFAR-10 and has a clean accuracy of 78.99%, which is lower than the ResNet and VGG models used in the main experiments. Second, although MSIS achieves the best average AUC, MSP slightly outperforms MSIS under the C&W attack. Third, the FPR@95TPR remains relatively high under FGSM and PGD, indicating that high-recall detection under transformer-style architectures remains challenging. Therefore, this experiment should be interpreted as a preliminary architectural stress test rather than comprehensive Transformer validation; systematic evaluation on ImageNet-scale datasets and larger Vision Transformer backbones remains future work.

To partially address the concern regarding sensor-related image artifacts, we further evaluate MSIS under several representative software-level image degradations on CIFAR-10 + ResNet-18. Specifically, four degradation types are considered: JPEG compression with quality factor 50, 4-bit quantization, additive Gaussian pixel noise with σ=0.02, and brightness reduction with a factor of 0.8. These degradations are applied consistently to both clean and adversarial samples in the same normalized evaluation pipeline.

As shown in Table 7, MSIS exhibits non-uniform sensitivity to different degradation types. Under 4-bit quantization, Gaussian pixel noise, and brightness reduction, the detector retains reasonable AUC performance, especially under PGD attacks. In contrast, JPEG compression causes a substantial performance drop, with the AUC decreasing to near-random levels across all three attacks. This suggests that lossy block-based compression may disrupt the fine-grained sensitivity responses used by MSIS for adversarial detection.

These results indicate that MSIS is not uniformly robust to all sensor-related image degradations. In particular, JPEG-compressed visual streams may require compression-aware feature normalization or a degradation-robust sensitivity metric. Therefore, this experiment provides a controlled software-level approximation of common sensor-related artifacts, complementing the broader scope discussion in the Conclusion.

#### 4.3.2. Robustness Against Different Attack Strengths and the Metric Inversion Phenomenon

More importantly, when facing strong iterative PGD attacks on the ResNet-50 model, baseline methods such as MSP [20] and Energy Score [21] exhibit abnormal and drastic AUC degradation (for example, the AUC of MSP on CIFAR-10 decreases to only 0.0203). This phenomenon is not caused by random fluctuation. Instead, strong iterative attacks tend to form highly confident stable regions in feature space, causing adversarial examples to exhibit anomalously low sensitivity under perturbations. Consequently, the decision function undergoes systematic directional inversion, where adversarial examples are consistently ranked ahead of normal samples.

This observation experimentally validates the “metric inversion” problem discussed in Section 2. Under a single-state metric framework, response patterns induced by different attacks may exhibit directional conflicts, thereby causing unified thresholds to fail. In contrast, the proposed method jointly models sensitivity features under both the native state and the temperature-smoothed state, enabling direction-adaptive correction in the extended feature space and effectively alleviating the metric inversion problem. Under the same setting, the proposed method achieves an AUC of 99.14% against PGD attacks, significantly outperforming all compared methods and further demonstrating the effectiveness and stability of dual-state feature modeling for handling heterogeneous attacks.

To further evaluate the robustness of the proposed detector under stronger and black-box attacks, additional experiments are conducted on CIFAR-10 with ResNet-18 using PGD-100 and Square Attack. PGD-100 uses the same perturbation budget as the original PGD setting, i.e., ϵ=8/255 and step size 2/255, but increases the number of optimization steps to 100. Square Attack is adopted as a query-based black-box attack with ϵ=8/255 and 5000 queries. Only adversarial examples that successfully induce misclassification are retained for detection evaluation.

As shown in Table 8, the proposed method maintains strong detection performance under both stronger white-box and black-box settings. Under PGD-100, MSIS achieves an AUC of 98.48% with an FPR@95TPR of 8.11%. Under Square Attack, MSIS achieves an AUC of 98.72% with an FPR@95TPR of 6.18%. These results indicate that the proposed detector remains effective when the attack strength is increased and when query-based black-box attacks are considered. Notably, the detection AUC under PGD-100 (98.48%) is slightly higher than that under PGD-10 (97.75%). This is consistent with our sensitivity-based interpretation that stronger iterative attacks may produce adversarial examples with more pronounced response variations under random perturbations, making them more distinguishable in the MSIS feature space.

Furthermore, all detector evaluation results reported in this section are obtained under the same stratified 5-fold cross-validation protocol, which helps reduce split-dependent evaluation bias. Under the non-intrusive setting where only output probability distributions are accessible, these results provide controlled benchmark evidence that MSIS maintains favorable detection performance across different attack strengths and access settings.

### 4.4. Mechanism Verification and Theoretical Consistency Analysis

To further investigate the underlying mechanisms of the proposed multi-domain fusion framework in handling heterogeneous attacks, this section analyzes the theoretical consistency of the MMP and DSF mechanisms from two perspectives: perturbation sensitivity in the spatial domain and response adaptation in the temperature-state domain. Experimental observations are further incorporated for validation.

#### 4.4.1. Spatial Domain: Revealing Local Manifold Vulnerability Through the MMP Mechanism

The essence of adversarial examples lies in exploiting the local nonlinearity of model decision boundaries [2]. Although optimization-based attacks such as C&W generate samples that are highly similar to natural samples in input space, these adversarial examples are often located in locally unstable regions in feature space and therefore exhibit strong response vulnerability [15]. The MMP mechanism is built upon the assumption that adversarial examples possess lower tolerance to random perturbations than natural samples [10], and characterizes this property through multi-scale perturbation probing.

**Figure 2:**
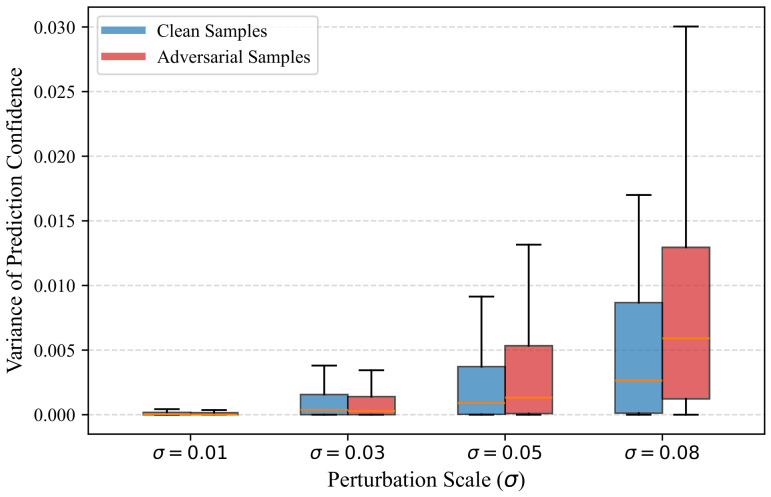
**Comparison of Prediction Confidence Variance Distributions Under Different Perturbation Scales**.

Experimental results validate the above assumption. As illustrated in Figure 2 (distribution of perturbation response variance), under small-scale random perturbations, the prediction outputs of natural samples remain relatively smooth, indicating that they reside in stable regions of the decision manifold. In contrast, the output responses of adversarial examples exhibit pronounced fluctuations as perturbation intensity increases. By jointly characterizing response variations under multiple perturbation scales, MMP effectively captures such local vulnerability characteristics and thereby enables preliminary discrimination of highly stealthy adversarial examples without relying on specific attack forms.

#### 4.4.2. Temperature-State Domain: Adaptive Correction of the Metric Inversion Phenomenon Through the DSF Mechanism

Under a single native state, strong iterative optimization attacks such as PGD shift adversarial examples toward high-confidence regions through multi-step optimization, causing their probability distributions to overlap with those of natural samples. In some regions, this even leads to reversal of the discrimination direction, thereby rendering detection methods based on single confidence metrics ineffective. This phenomenon is also validated in the main experiments of this paper: on the ResNet-50 model, the detection performance of MSP and Energy Score decreases significantly under PGD attacks, indicating the existence of a typical metric inversion problem.

To alleviate this issue, the proposed DSF mechanism introduces a temperature-smoothed state [7]. By adjusting the smoothness of the output distribution, hidden response differences can be more effectively revealed. Under high-temperature conditions, the overconfidence of adversarial examples is suppressed, gradually exposing their intrinsic sensitivity to input perturbations. In contrast, natural samples exhibit more stable response patterns across different temperature states.

**Figure 3:**
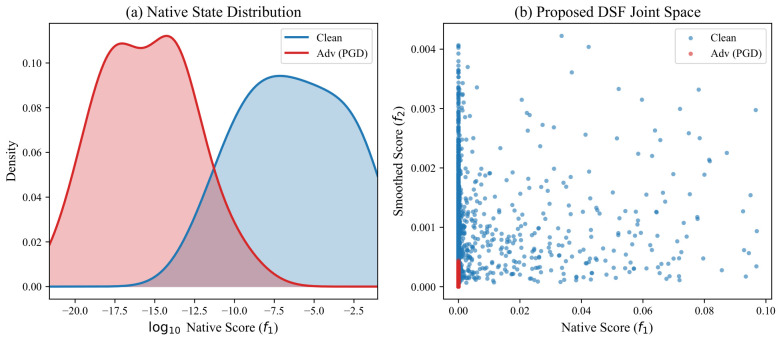
**Comparison of Sample Distributions in Native-State and Dual-State Feature Spaces**.

From the perspective of feature-space mapping, DSF essentially expands the original low-dimensional probability responses into a joint feature representation through the introduction of multiple temperature states. As illustrated in Figure 3 (visualization of dual-state joint feature distributions), samples that exhibit severe overlap under a single state become more clearly separable in the dual-state feature space. Further analysis of the linear classifier weights indicates that the model can learn differentiated weight assignments across different state features, thereby enabling adaptive correction of abnormal response directions.

In summary, the MMP mechanism characterizes the local manifold vulnerability of adversarial examples from the perspective of the spatial domain, while the DSF mechanism alleviates the metric inversion problem caused by heterogeneous attacks through temperature-state-domain feature expansion. The two mechanisms enhance feature discriminability from complementary perspectives, and their synergistic interaction jointly constructs a stable joint feature space. Experimental observations further validate the theoretical analysis presented in Section 2 regarding decision manifold characteristics and attack distribution heterogeneity.

### 4.5. Ablation Study

To evaluate the individual effectiveness and synergistic contribution of each core component in the proposed multi-domain fusion framework, ablation experiments are conducted on the CIFAR-10 dataset using the ResNet-18 model. The results are presented in Table 9.

**(1) Analysis of Individual Module Effectiveness**

Experimental results show that introducing the spatial-domain perturbation mechanism (MMP) alone can substantially improve detection performance. Taking PGD attacks as an example, the AUC increases from 65.77% for the baseline method to 91.02%, and further reaches 96.19% under C&W attacks. These results indicate that multi-scale random perturbations can effectively amplify the local manifold vulnerability of adversarial examples, making their anomalous responses in feature space more distinguishable and thereby improving inter-class separability.

In contrast, when only the temperature-state dual-feature mechanism (DSF) is introduced, the overall detection performance remains relatively limited (AUC approximately ranging from 53% to 72%). The fundamental reason is that DSF does not directly enhance spatial separability between samples. Instead, by introducing a temperature-smoothed state [7], it recalibrates the confidence distribution of model outputs and alleviates the abnormal responses and metric inversion issues caused by strong iterative attacks such as PGD. Consequently, under PGD attacks, DSF still achieves a certain degree of improvement compared with the baseline method (65.77% → 72.03%), validating its effectiveness in temperature-state-domain correction.

**(2) Analysis of Synergistic Effects Between Modules**

When MMP and DSF are jointly integrated, the detection performance reaches the best level across all attack scenarios. Specifically, the AUC values for FGSM, PGD, and C&W attacks reach 97.01%, 97.75%, and 97.72%, respectively, while FPR@95TPR is significantly reduced to the range of 7.75–13.54%.

These results indicate that MMP and DSF do not simply contribute additive performance gains, but instead form a collaborative mechanism with complementary functional roles. MMP actively injects multi-scale perturbations into the input space to explicitly amplify the local manifold vulnerability of adversarial examples. DSF, on the other hand, reconstructs model responses in the temperature-state domain and effectively corrects metric direction deviations that may arise under a single state through joint modeling of multi-temperature features.

From the perspective of feature-space representation, MMP provides highly sensitive spatial response signals, while DSF further decouples and aligns these signals within an expanded state space. As a result, samples that are difficult to separate under a single-dimensional representation become more distinguishable in the joint feature space with stable decision boundaries. The deep integration of these two mechanisms fundamentally improves the robustness of the proposed framework against heterogeneous attacks.

**(3) Analysis of Logistic Fusion Weights**

To further examine the role of the linear fusion classifier, we analyze the learned logistic-regression coefficients under the full MSIS feature space. The 9-dimensional feature vector consists of one temperature-state discrepancy feature, four native-state multi-scale sensitivity features, and four temperature-smoothed multi-scale sensitivity features. The learned coefficients show attack-dependent weighting patterns. For FGSM and PGD, the largest positive coefficients are mainly assigned to temperature-smoothed multi-scale features, especially T=5,σ=0.02 and T=5,σ=0.08. In contrast, for C&W attacks, the native-state small-scale feature (T=1,σ=0.01) receives the largest positive coefficient, while the native-state medium-scale feature (T=1,σ=0.05) obtains a negative mean coefficient and is negative in four out of five folds. These results indicate that the linear classifier does not simply aggregate all sensitivity features in the same direction, but learns attack-dependent state-scale weighting patterns, providing quantitative evidence for state-dependent correction in the DSF feature space.

**(4) Analysis of Temperature Parameter Selection in DSF**

To validate the rationality of the smoothing temperature parameter ($T_{2}$) in the DSF module, experiments are conducted with MMP disabled, so as to isolate the independent influence of temperature-state smoothing. Therefore, Table 10 should be interpreted as a controlled analysis of the DSF temperature parameter rather than the full MSIS performance. In this setting, $T_{1}$ is fixed to 1, while systematic ablation experiments are performed with: $T_{2}\in \left\{1,2,5,10,20\right\}$. The corresponding results are presented in Table 10. After this ablation, $T_{2}=5$ is fixed as the default setting and is not re-tuned for different attacks or held-out test samples.

Experimental results show that when $T_{2}=1$ (i.e., the two temperature branches are identical), the features extracted from the native state and the smoothed state become highly homogeneous, providing limited complementary information. Under this setting, the average AUC across the three attack types reaches only 0.9232. As $T_{2}$ increases, the temperature smoothing operation [7] progressively suppresses the artificially high confidence associated with strong iterative attacks, thereby enhancing the complementarity between dual-state features and continuously improving the AUC performance.

The optimal average AUC (0.9492) is achieved when $T_{2}=5$, where FPR@95TPR also reaches its minimum value. However, when $T_{2}$ is further increased ($T_{2}=10,20$), excessive smoothing causes the output distribution to become overly uniform, diluting effective response signals and resulting in performance degradation. Therefore, this paper ultimately adopts $T_{2}=5$ as the smoothing temperature parameter of the DSF module, which is supported by clear experimental evidence.

Since the above temperature ablation isolates the DSF module by disabling MMP, we further evaluate the stability of different $T_{2}$ values under the complete MSIS framework. In this full-model evaluation, MMP remains enabled, the probing scales are fixed to {0.01,0.02,0.05,0.08}, and the same 9-dimensional MSIS feature construction and 5-fold cross-validation protocol are used.

As shown in Table 11, the complete MSIS framework is not highly sensitive to the exact choice of $T_{2}$ within a reasonable range. When $T_{2}$ increases from 5 to 20, the average AUC remains stable between 97.48% and 97.53%, indicating that the proposed detector does not rely on a fragile temperature setting. The lower performance at $T_{2}=2$ suggests that insufficient smoothing provides weaker complementary temperature-state information. Notably, the increase in FPR@95TPR at $T_{2}=2$ (e.g., from 13.40% to 21.24% for FGSM) is proportionally larger than the corresponding AUC drop, suggesting that insufficient temperature smoothing primarily degrades reliability under high-recall operating conditions. Therefore, $T_{2}=5$ is retained as the default setting because it provides strong overall performance and relatively low FPR@95TPR while keeping the configuration simple and fixed across attacks.

**(5) Analysis of Distance Metrics and Noise Distributions**

In addition to the temperature parameter, we further evaluate the influence of different distance metrics and perturbation noise distributions. This ablation is further expanded in the current revision with additional discussion of the rationale behind the default $L_{1}$ + Gaussian configuration. In this ablation, clean and adversarial samples are paired by retaining only successfully misclassified adversarial examples and their corresponding clean counterparts. Therefore, the absolute AUC values may slightly differ from those in the main comparison table, while the relative comparisons among different metrics and noise distributions remain valid. For the KL-based variant, we compute the asymmetric divergence in the direction from the original output distribution to the perturbed output distribution.

As shown in Table 12, under Gaussian perturbations, the $L_{1}$ metric achieves the highest average AUC (98.59%) and lower FPR@95TPR than $L_{2}$ and KL on FGSM and PGD attacks. Replacing $L_{1}$ with $L_{2}$ decreases the average AUC to 98.15% and increases FPR@95TPR, indicating that $L_{1}$ is more suitable for capturing sparse probability-response variations induced by local perturbations. KL divergence also performs competitively, but it does not outperform $L_{1}$ and is direction-sensitive by definition. Regarding the perturbation distribution, uniform and Laplace perturbations produce similar AUC values to Gaussian perturbations, suggesting that MSIS is not overly sensitive to a specific random noise distribution. Although $L_{1}$ + Laplace obtains a similar average AUC, $L_{1}$ + Gaussian provides more stable FPR@95TPR across FGSM and PGD. Therefore, Gaussian perturbations are retained as the default because they are consistent with the isotropic local probing assumption used in the MSIS formulation and provide stable performance across heterogeneous attacks.

**(6) Analysis of the Number of Probing Scales**

We further evaluate the influence of the number of probing scales on detection performance. Similar to the metric and noise ablations, this experiment uses paired clean and adversarial samples by retaining only successfully misclassified adversarial examples and their corresponding clean counterparts. The 2-scale, 4-scale, and 6-scale settings correspond to feature dimensions of 5, 9, and 13, respectively.

As shown in Table 13, increasing the number of probing scales from 2 to 4 improves the average AUC from 98.44% to 98.58% and reduces FPR@95TPR across attacks. Further increasing the number of scales to 6 provides only marginal additional improvement in average AUC (98.59%) while increasing the feature dimensionality and computational cost. Therefore, the 4-scale setting provides a favorable trade-off between detection performance and efficiency, and is adopted as the default configuration.

**(7) Analysis of Fusion Classifier Choices**

To examine whether the linear logistic-regression fusion classifier limits the discriminative capability of DSF, we further compare it with several nonlinear fusion classifiers, including a multi-layer perceptron (MLP), Random Forest, and RBF-kernel SVM. All classifiers are evaluated using the same 9-dimensional MSIS feature vector, the same stratified 5-fold cross-validation protocol, and the same CIFAR-10 + ResNet-18 setting.

As shown in Table 14, nonlinear fusion classifiers do not provide substantial additional improvement over logistic regression. The MLP achieves the highest average AUC of 97.69%, but the improvement over logistic regression is only 0.17 percentage points. Random Forest performs almost identically to logistic regression, while RBF-SVM slightly decreases the average AUC. These results suggest that the 9-dimensional MSIS feature representation is already highly discriminative, and that a simple linear decision boundary is sufficient for effective fusion in this feature space. Therefore, logistic regression is retained as the default fusion classifier due to its interpretability, low computational overhead, and stable performance.

### 4.6. Adaptive Attack Evaluation and Stress Test

To further evaluate the robustness of the proposed method under strong adversarial threat models, we conduct two complementary detector-evasion stress tests. First, adaptive attacks [24] are constructed under a white-box EoT-based setting, where the attacker is assumed to have complete knowledge of the internal details of the detection framework, including the construction mechanisms of multi-scale perturbation probing (MMP) and dual-state features (DSF). In this setting, the attacker attempts to evade the detector through a joint optimization objective that balances classification disruption and sensitivity suppression. The corresponding white-box EoT-based results are presented in Table 15.

Second, to examine whether detector-evasion behavior can transfer across independently trained detectors, we further conduct a surrogate-detector transfer attack. In this setting, the attacker trains a separate surrogate MSIS detector using a different random seed and a limited training subset, optimizes adversarial examples against the surrogate detector score, and then evaluates the generated samples using the true MSIS detector. This experiment is designed to assess black-box detector-level transferability rather than full white-box access to the true detector.

#### 4.6.1. Adaptive Attack Modeling

In traditional adversarial attacks, the objective is typically only to maximize classification error. In contrast, under an adaptive attack scenario, the attacker must achieve misclassification while simultaneously minimizing the discriminative response of the detector. To this end, this paper constructs the following joint optimization objective:

(11) $$\min_{\delta }\;L_{\mathrm{margin}}\left(x+\delta ,y\right)+\lambda \cdot S_{\mathrm{sens}}\left(x+\delta \right),\;{∥\delta ∥}_{\infty }\leq \epsilon$$

where $L_{\mathrm{margin}}$ denotes a CW-style classification margin loss defined as $\max(f_{y}\left(x+\delta \right)-\max_{j\neq y}f_{j}\left(x+\delta \right),-\kappa )$, $S_{\mathrm{sens}}$ denotes the EoT-estimated perturbation-sensitivity penalty used to suppress the detector-related sensitivity response, and λ is a trade-off parameter regulating the attack objective’s bias between “disrupting classification” and “evading detection.”

Since MSIS features rely on a random perturbation sampling process, direct optimization suffers from gradient instability. Therefore, this paper introduces the Expectation over Transformation (EoT) technique [25] to approximate the expectation over random perturbations, thereby obtaining a stable and differentiable optimization objective:

(12) $$S_{\mathrm{sens}}\left(x^{\prime }\right)=E_{\sigma \in \Sigma ,\;\epsilon ∼N(0,\sigma^{2}I)}\left[{∥f\left(x^{\prime }\right)-f\left(x^{\prime }+\epsilon \right)∥}_{1}\right].$$

In implementation, the expectation is approximated by averaging over $K_{\mathrm{eot}}=3$ Monte Carlo samples for each probing scale in Σ. The perturbation δ is optimized using the Adam optimizer with step size $\alpha_{\mathrm{lr}}=0.01$ and 100 optimization steps, and projected onto the $L_{\infty }$ ball of radius ϵ=8/255 after each step.

#### 4.6.2. Experimental Results and Trade-Off Analysis

This paper conducts a systematic scan over different trade-off parameters λ on the CIFAR-100 dataset [17] using the ResNet-18 model [19], extending the evaluation to a log-scaled range of λ∈{0,1,3,5,10,30,100,300,1000} to map out the operational behavior of MSIS under increasing evasion pressure.

Experimental results reveal a non-monotonic trade-off pattern. When λ increases from 0 to 10, the detection AUC decreases from 96.75% to 80.54%, while the ASR remains high (above 92%), indicating that moderate evasion pressure can effectively weaken the detector without sacrificing misclassification capability. However, as λ further increases beyond 10, a clear reversal is observed: the ASR drops substantially from 92.40% at λ=10 to 52.30% at λ=1000, while the detection AUC recovers to approximately 87%. This indicates that beyond a certain evasion pressure threshold, the attack objective becomes dominated by the sensitivity penalty, causing the adversarial optimization to sacrifice misclassification capability in order to suppress the detection score.

Within the tested EoT-based adaptive protocol, these results suggest that maintaining high ASR and low detection AUC simultaneously becomes difficult across the full λ range. The observed trade-off pattern is consistent with the interpretation that the multi-domain coupling of MMP and DSF introduces non-trivial optimization constraints under this adaptive setting. Nevertheless, we acknowledge that the detector performance is clearly weakened in the moderate λ regime (λ∈[1,10]), where ASR remains high while AUC drops to approximately 80%.

#### 4.6.3. Surrogate-Detector Transfer Attack

To further examine whether attacks against the detector can transfer across independently trained MSIS detectors, we conduct a surrogate-detector transfer attack on CIFAR-10 + ResNet-18. In this setting, the true MSIS detector is trained using the defender-side data split, while a surrogate MSIS detector is trained with a different random seed and only half of the defender training samples. The attacker does not directly optimize against the true detector; instead, it optimizes adversarial examples against the surrogate detector score and then evaluates the generated samples using the true detector.

This experiment should be interpreted as a detector-level transfer stress test rather than the same EoT-based adaptive protocol used in Table 15. Therefore, the λ=0 result serves as the internal baseline of this transfer-attack setting and is not directly comparable to the main results in Table 2.

As shown in Table 16, the detector-level transfer attack substantially weakens MSIS. When λ increases from 0 to 0.0003, the true detector AUC decreases from 91.08% to 63.09% while the ASR remains 100%. Under stronger detector-evasion pressure (λ=0.001 and 0.002), the AUC further drops below random-level performance, indicating that the surrogate detector score can be transferred to the true detector and exploited to invert the detection tendency. These results reveal an important limitation of MSIS: although the method performs well under standard and EoT-based benchmark attacks, detector-score-aware transfer attacks can substantially reduce its effectiveness. Developing training or calibration strategies against such detector-level transfer attacks is therefore an important direction for future work.

#### 4.6.4. Mechanism Analysis and Robustness Discussion

The above results show that the proposed MSIS framework retains partial detection capability under the tested adaptive attack setting, but its performance is clearly weakened as the evasion pressure increases. This indicates that MSIS is not fully robust against a knowledgeable adaptive attacker, and the adaptive attack results should be interpreted as a robustness stress test rather than a proof of robustness.

The observed trade-off may be related to the multi-domain coupling characteristics of the detection features:

- **Spatial Domain (MMP):** Multi-scale perturbation probing characterizes response variations under different noise scales, which may increase the difficulty of simultaneously maintaining adversarial misclassification and low sensitivity responses.
- **Temperature-State Domain (DSF):** Temperature-state feature expansion introduces additional discriminative dimensions, which may make detector evasion more difficult when the attacker attempts to suppress abnormal responses across multiple model response states.

Intuitively, the classification loss and the sensitivity penalty tend to impose competing optimization pressures: reducing sensitivity often requires the adversarial example to reside in a stable, flat region of the feature distribution, which conflicts with the boundary-crossing requirement for successful misclassification. This tension may contribute to the observed trade-off between ASR and detection AUC. However, we acknowledge that this argument is presented as an intuitive explanation rather than a formal proof, and stronger adaptive attacks with more optimization steps may further challenge the proposed detector.

The reversal observed when λ>10 is consistent with this interpretation: excessive evasion pressure appears to dominate the optimization objective, reducing the attack success rate while no longer further suppressing the detector response.

### 4.7. Failure-Case Analysis

To better understand the limitations of MSIS, we further analyze typical missed detections and false positives. Missed adversarial examples usually correspond to samples whose output probability distributions remain stable under random perturbations. Such samples may lie in locally flat regions of the decision surface after successful adversarial optimization, making their multi-scale sensitivity responses similar to those of clean samples. This failure mode is especially likely for strong iterative attacks that produce highly confident adversarial predictions.

False positives, on the other hand, are often associated with clean samples located near class boundaries or samples with intrinsically ambiguous visual patterns. For these samples, small random perturbations may induce noticeable changes in the output probability vector, causing their sensitivity features to resemble those of adversarial examples. This phenomenon is more pronounced in high-density classification tasks such as CIFAR-100, where inter-class decision regions are more compressed.

These failure modes are consistent with the quantitative observations in Section 4.3. Under the CIFAR-100 + ResNet-18 configuration, MSIS achieves an average AUC of 82.60%, notably lower than the 94.17% under CIFAR-100 + ResNet-50. Similarly, under adaptive attacks, the detection AUC decreases to 80.54% at λ=10 (Table 15), confirming that explicit evasion pressure can meaningfully weaken the detector. The surrogate-detector transfer results in Table 16 further show that detector-score-aware transfer attacks may cause even stronger degradation when the attacker optimizes against a transferable surrogate detector.

These observations indicate that MSIS is most effective when adversarial examples introduce detectable instability in the output probability space, but it may fail when adversarial examples remain locally stable or when clean samples are naturally close to decision boundaries. This limitation is consistent with the output-probability-based nature of the proposed detector and motivates future investigation of richer feature representations. Table 17 summarizes the main failure patterns and their corresponding interpretations.

### 4.8. Practical Limitations and Mitigation Strategies

Although MSIS achieves strong AUC performance under the evaluated benchmark settings, its FPR@95TPR remains non-negligible in several cases. For example, under the CIFAR-10 + ResNet-18 configuration, the FPR@95TPR of the full MSIS detector ranges from 7.75% to 13.54% across FGSM, PGD, and C&W attacks. This indicates that MSIS should not be interpreted as a deployment-ready detector for safety-critical real-time systems where extremely low false-alarm rates are required.

A concrete mitigation strategy is to introduce cost-sensitive operating-point calibration. Instead of using a fixed threshold directly derived from ROC analysis, the detection threshold can be selected on a validation set by explicitly minimizing a deployment-specific cost function, such as

(13) $$\gamma^{*}=\arg\min_{\gamma }\left(c_{\mathrm{FP}}\mathrm{FPR}\left(\gamma \right)+c_{\mathrm{FN}}\mathrm{FNR}\left(\gamma \right)\right),$$

where $c_{\mathrm{FP}}$ and $c_{\mathrm{FN}}$ denote the relative costs of false positives and false negatives. In safety-critical monitoring scenarios, this calibration allows the operating point to be adjusted according to application-specific tolerance for false alarms and missed detections.

Another possible mitigation direction is degradation-aware or attack-aware calibration. The simulated sensor-level degradation experiment shows that compression, quantization, noise, and brightness changes affect MSIS differently. Therefore, separate calibration layers or validation profiles can be constructed for different input conditions, such as clean digital inputs, compressed image streams, or noise-corrupted visual data. A lightweight post-hoc calibration model, such as isotonic regression or Platt scaling, could be fitted on top of the logistic-regression detection score to improve threshold reliability under each condition.

For reducing computational overhead, a concrete acceleration route is feature-level distillation. A lightweight student detector can be trained to approximate the MSIS score produced by the full multi-scale probing procedure:

(14) $$L_{\mathrm{distill}}={∥g_{\theta }\left(p\left(x\right)\right)-s_{\mathrm{MSIS}}\left(x\right)∥}_{2}^{2},$$

where p(x) denotes the output probability vector of the target classifier, $s_{\mathrm{MSIS}}\left(x\right)$ denotes the teacher score obtained from the full MSIS procedure, and $g_{\theta }$ is a lightweight student model. Such a student detector could reduce the number of forward passes required during inference while preserving part of the discriminative information learned from MSIS.

Finally, intermediate-layer optimization may provide richer detection features, but it would require access to internal model activations and therefore weaken the non-intrusive nature of MSIS. For this reason, the current study keeps the detector strictly output-probability-based. We regard cost-sensitive calibration, degradation-aware score calibration, and feature-level distillation as concrete future directions for reducing FPR@95TPR and improving practical deployability.

### 4.9. Computational Efficiency and Practical Overhead Analysis

To further evaluate the computational costs of different detection methods, this paper measures the per-sample inference latency under the same benchmark hardware/software environment (CIFAR-10 + ResNet-18, GPU: CUDA). The results are shown in Table 18. This analysis focuses on computational overhead under controlled benchmark settings rather than embedded-hardware deployment readiness.

Experimental results indicate that single-forward-pass methods, such as MSP [20] and Energy Score [21], exhibit the lowest inference latency (approximately 0.1 ms/sample). The inference latency of MSIS is 4.076 ms/sample, which is approximately 40 times that of these single-score baselines. This overhead mainly comes from the multi-scale Monte Carlo probing process and the dual-temperature feature extraction mechanism, which require approximately 80 forward passes in total.

Therefore, MSIS should not be interpreted as a real-time edge detector for high-frequency sensor streams. The measured latency of 4.076 ms/sample is obtained under the benchmark hardware/software environment used in this study and should not be directly extrapolated to embedded platforms such as NVIDIA Jetson devices. Nevertheless, this latency remains practically feasible for latency-tolerant batch-level inspection, offline security auditing, and non-intrusive model evaluation scenarios where only output probabilities are available. Under this setting, the additional latency trades off for stronger detection performance and lower access requirements: MSIS improves the average AUC from approximately 0.80 for single-score baselines to 0.9749, while requiring no access to model gradients, intermediate features, or network parameters.

Compared with PASA [23], which requires gradient access and incurs an inference latency of 47.344 ms/sample, MSIS achieves substantially lower latency while remaining non-intrusive. Specifically, MSIS requires only output probabilities and achieves an inference latency of 4.076 ms/sample, approximately 1/11th that of PASA. These results indicate that MSIS provides a favorable trade-off between detection performance, access requirements, and computational overhead under the evaluated benchmark setting. Nevertheless, embedded deployment would require additional lightweight optimization and direct latency and energy profiling on target hardware platforms.

## 5. Conclusions and Future Work

In response to adversarial threats against deep neural networks in safety-critical visual classification tasks [2], this paper proposes and systematically evaluates the Multi-scale Input Sensitivity (MSIS) framework for adversarial example detection. MSIS combines task-level multi-scale perturbation probing with temperature-state feature modeling to improve the discriminability of output-probability responses under heterogeneous attacks. By relying only on output probabilities, the framework provides a non-intrusive detection strategy without modifying the original network weights or accessing internal gradients. Additional white-box adaptive evaluation [24] suggests that the multi-domain fusion framework introduces competing optimization objectives between classification disruption and detector evasion, although stronger adaptive evaluations remain necessary for fully characterizing the robustness boundary of the proposed method. Moreover, the surrogate-detector transfer experiment shows that detector-score-aware transfer attacks can substantially reduce the detection AUC, indicating that black-box detector-level evasion remains an important open challenge for MSIS.

However, this study still presents several limitations. First, due to the reliance on multiple forward passes and Monte Carlo sampling, the computational overhead and latency during inference increase compared with single-forward-pass detectors. Therefore, the current implementation is more suitable for offline security auditing and batch-level adversarial sample screening than for real-time edge deployment. Second, although additional experiments with PGD-100, Square Attack, VGG-16-BN, and a lightweight ViT backbone are included, the current evaluation is still mainly conducted on CIFAR-10 and CIFAR-100 with ResNet-18 and ResNet-50 under a fixed 5-fold cross-validation protocol. The VGG-16-BN and lightweight ViT experiments should be viewed as limited architectural stress tests under CIFAR-10 rather than comprehensive validation across modern architectures. Although an additional repeated-run analysis over five random seeds is provided on the CIFAR-10 + ResNet-18 configuration, broader statistical evaluation across more datasets, architectures, and attack settings remains an important direction for future work. Evaluation on ImageNet-scale datasets, larger Vision Transformer architectures [26,27], and broader modern convolutional backbones such as ConvNeXt [28] remains an important direction for future work. Third, although we have added a software-level degradation analysis involving JPEG compression, quantization, Gaussian pixel noise, and brightness variation, real sensor artifacts have not yet been systematically modeled in an end-to-end physical sensor pipeline. The current study should be viewed as an algorithmic investigation of output-probability-based adversarial detection under controlled benchmark settings, rather than a deployment-ready solution for real-time sensor systems. Embedded hardware validation, including latency and energy profiling on platforms such as NVIDIA Jetson devices, sensor-specific noise modeling, and end-to-end perception-pipeline evaluation remain important directions for broader system-level studies. Extending the proposed sensitivity-based detection framework from image classification to object detection is also worth further investigation, since adversarial attacks against object detectors introduce additional localization and proposal-level vulnerabilities [29].

To address the aforementioned bottlenecks, future research may explore three concrete directions. First, cost-sensitive threshold calibration or post-hoc score calibration could be used to reduce FPR@95TPR under specific deployment requirements. Second, feature-level distillation [30] could train a lightweight student detector to approximate the full MSIS score, thereby reducing the number of forward passes required by multi-scale Monte Carlo probing. Third, intermediate-layer sensitivity features may further improve discriminability, but they would require access to internal activations and therefore weaken the non-intrusive assumption of the current framework. The supplementary fusion-classifier comparison further confirms that the compact 9-dimensional MSIS feature representation is largely linearly separable, supporting the use of logistic regression as an interpretable and sufficiently effective default choice. The additional metric-and-noise ablation further shows that the default $L_{1}$ + Gaussian configuration provides a stable trade-off among detection performance, FPR@95TPR, and consistency with the local perturbation-probing formulation. The full-MSIS temperature stability analysis further indicates that the detector is not highly sensitive to the exact choice of $T_{2}$ within a reasonable range, although adaptive temperature selection remains an interesting direction for future investigation.

## Figures and Tables

**Table 1:** Adversarial Attack Parameter Settings and Attack Success Rates.

Attack	Core Parameters	CIFAR-10		CIFAR-100	
		R18	R50	R18	R50
FGSM	ϵ=8/255	57.66%	86.85%	79.65%	98.95%
PGD	ϵ=8/255, α=2/255, steps = 10	80.67%	100.00%	96.80%	99.15%
C&W	c=1, steps = 100, lr = 0.01	92.68%	100.00%	100.00%	99.00%

**Table 2:** AUC Evaluation Results of Different Detection Methods.

Dataset	Target Model	Detection Method	FGSM	PGD	C&W	Average
CIFAR-10	ResNet-18	MSP	0.7755	0.6681	0.8754	0.7730
		Feature Squeezing	0.8245	0.7220	0.6330	0.7265
		Energy Score	0.8178	0.7343	0.8510	0.8010
		DBA-style Reconstruction	1.0000	1.0000	0.4879	0.8293
		Mahalanobis	0.5508	0.5392	0.5075	0.5325
		PASA	0.7601	0.6843	0.9160	0.7868
		**MSIS (Ours)**	**0.9701**	**0.9775**	**0.9772**	**0.9749**
	ResNet-50	MSP	0.8568	0.9797 ^*^	0.8750	0.9038
		Feature Squeezing	0.7777	0.9759 ^*^	0.8933	0.8823
		Energy Score	0.8613	0.9878 ^*^	0.8026	0.8839
		DBA-style Reconstruction	0.6387	0.5521	0.5040	0.5649
		Mahalanobis	0.8485	0.9771	0.6290	0.8182
		PASA	0.8170	0.6233	0.6284	0.6896
		**MSIS (Ours)**	**0.9843**	**0.9914**	**0.9050**	**0.9602**
CIFAR-100	ResNet-18	MSP	0.7808	0.6050	0.8479	0.7446
		Feature Squeezing	0.7083	0.5220	0.8075	0.6793
		Energy Score	0.7770	0.6453	0.7420	0.7214
		DBA-style Reconstruction	0.5075	0.5018	0.5027	0.5040
		Mahalanobis	0.6582	0.6337	0.6282	0.6401
		PASA	0.5498	0.5852	0.5361	0.5570
		**MSIS (Ours)**	**0.8790**	**0.7595**	**0.8394**	**0.8260**
	ResNet-50	MSP	0.8478	0.9222 ^*^	0.8509	0.8736
		Feature Squeezing	0.8800	0.9661 ^*^	0.7486	0.8649
		Energy Score	0.6919	0.9290 ^*^	0.8284	0.8164
		DBA-style Reconstruction	0.6136	0.5376	0.5029	0.5514
		Mahalanobis	0.8200	0.9697	0.5561	0.7819
		PASA	0.8155	0.5595	0.5079	0.6276
		**MSIS (Ours)**	**0.9870**	**0.9832**	**0.8548**	**0.9417**

^*^ indicates that severe score-direction inversion is observed for the corresponding single-score baseline. The reported value is obtained after applying the fold-wise direction-selection rule described in Section 4.1.3, where the score direction is determined only from the training split and then fixed for evaluation on the held-out split. Bold values indicate the best performance within each comparison block.

**Table 3:** Model Access Requirements of Different Detection Methods.

Detection Method	MSP	Feature Squeezing	Energy Score	DBA-Style Reconstruction	Mahalanobis	PASA	MSIS (Ours)
Access Requirement	Non-intrusive	Non-intrusive	Non-intrusive	Black-box	Intrusive	Intrusive	Non-intrusive

**Table 4:** Repeated-run stability analysis of MSIS on CIFAR-10 + ResNet-18 over five random seeds. Results are reported as mean ± standard deviation in percentage.

Attack	AUC (%)	FPR@95TPR (%)
FGSM	97.04±0.02	13.35±0.11
PGD	97.77±0.02	11.05±0.05
C&W	97.75±0.02	7.55±0.17

**Table 5:** Additional Architectural Generalization Evaluation on CIFAR-10 Using VGG-16-BN.

Detection Method	FGSM	PGD	C&W	Average
MSP	0.7691	0.7880	0.8891	0.8154
Energy Score	0.7662	0.8693	0.8583	0.8313
**MSIS (Ours)**	**0.8054**	**0.9031**	**0.9054**	**0.8713**

**Table 6:** Preliminary Architectural Stress Test on CIFAR-10 Using a Lightweight ViT Backbone (AUC/FPR@95TPR, %).

Detection Method	FGSM	PGD	C&W	Average AUC
MSP	54.10/94.48	66.03/70.20	89.14/29.20	69.76
Energy Score	58.48/89.37	55.74/80.60	69.43/73.90	61.22
MSIS (Ours)	78.42/67.02	80.04/60.00	87.35/28.20	81.94

**Table 7:** MSIS Detection Performance Under Simulated Sensor-Level Degradations on CIFAR-10 + ResNet-18 (AUC/FPR@95TPR, %).

Degradation	FGSM	PGD	C&W
None (baseline)	93.42/30.42	99.02/4.40	88.98/38.00
JPEG (q = 50)	62.85/85.83	52.98/91.40	51.07/94.20
4-bit Quantization	90.20/39.58	98.52/6.60	82.02/47.20
Gaussian Noise (σ=0.02)	89.20/51.25	98.76/5.80	79.11/51.20
Brightness (×0.8)	92.92/35.42	98.91/3.20	87.34/37.20

**Table 8:** Additional Evaluation Under Stronger White-box and Black-box Attacks on CIFAR-10 + ResNet-18.

Attack	Attack Type	ASR (%)	AUC/FPR@95TPR (%)
PGD-100	Strong white-box iterative	85.10	98.48/8.11
Square Attack	Query-based black-box	79.30	98.72/6.18

**Table 9:** Ablation Results of Core Components in the Multi-domain Fusion Framework (AUC/FPR@95TPR, %).

Variant	MMP	DSF	FGSM	PGD	C&W
Baseline (MSP)	×	×	77.15/59.37	65.78/78.89	87.66/44.02
w/o DSF (MMP Only)	✓	×	91.00/43.80	90.90/43.54	96.18/18.52
w/o MMP (DSF Only)	×	✓	63.73/79.01	72.03/71.04	53.87/80.76
**MSIS (Ours)**	✓	✓	**97.01/13.54**	**97.75/11.11**	**97.72/7.75**

Bold values indicate the best performance. The symbols ✓ and × indicate whether the corresponding module is enabled or disabled.

**Table 10:** Ablation Results of the Temperature Parameter ($T_{2}$) in the DSF Module (AUC/FPR@95TPR, %).

*T*_1_	*T*_2_	FGSM	PGD	C&W	Average AUC
1	1	0.9086/44.35%	0.8999/42.40%	0.9611/19.95%	0.9232
1	2	0.9302/37.80%	0.9294/36.50%	0.9670/13.90%	0.9422
1	5	0.9378/30.25%	0.9395/29.85%	0.9702/11.75%	**0.9492**
1	10	0.9272/34.25%	0.9237/34.10%	0.9671/15.55%	0.9394
1	20	0.9204/37.95%	0.9146/37.25%	0.9650/17.30%	0.9333

Bold values indicate the best average AUC.

**Table 11:** Full-MSIS temperature stability analysis on CIFAR-10 + ResNet-18 (AUC/FPR@95TPR, %).

*T*_2_	FGSM	PGD	C&W	Average AUC
2	95.69/21.24	96.63/17.00	97.33/8.68	96.55
5	97.03/13.40	97.78/10.95	97.75/7.42	97.52
10	97.00/14.00	97.76/10.88	97.83/7.41	97.53
20	96.96/14.17	97.66/11.60	97.82/7.27	97.48

**Table 12:** Ablation Results of Distance Metrics and Noise Distributions on CIFAR-10 + ResNet-18 (AUC/FPR@95TPR, %).

Configuration	FGSM	PGD	C&W	Average AUC
*L*_1_ + Gaussian	98.26/8.00	98.52/7.46	98.99/5.06	98.59
*L*_2_ + Gaussian	97.74/10.24	97.96/11.04	98.75/5.33	98.15
KL + Gaussian	98.14/8.95	98.39/8.32	98.82/5.70	98.45
*L*_1_ + Uniform	98.04/9.38	98.39/8.08	98.94/4.90	98.46
*L*_1_ + Laplace	98.26/8.69	98.52/8.75	99.00/4.79	98.59

**Table 13:** Ablation Results of the Number of Probing Scales on CIFAR-10 + ResNet-18 (AUC/FPR@95TPR, %).

Scale Setting	Feature Dim.	FGSM	PGD	C&W	Average AUC
2 scales {0.01,0.05}	5	98.08/9.38	98.29/8.57	98.94/4.79	98.44
4 scales {0.01,0.02,0.05,0.08}	9	98.24/8.35	98.46/8.08	99.03/4.63	98.58
6 scales {0.005,0.01,0.02,0.05,0.08,0.10}	13	98.22/8.35	98.49/7.83	99.06/4.25	98.59

**Table 14:** Comparison of different fusion classifiers on CIFAR-10 + ResNet-18 (AUC/FPR@95TPR, %).

Fusion Classifier	FGSM	PGD	C&W	Average AUC
Logistic Regression	97.03/13.40	97.78/10.95	97.75/7.42	97.52
MLP	97.20/13.07	97.84/10.97	98.02/6.86	97.69
Random Forest	97.12/13.08	97.79/10.57	97.71/8.28	97.54
RBF-SVM	95.88/12.77	96.90/10.63	97.03/6.78	96.60

**Table 15:** Trade-off Between Attack Success Rate and Detection AUC Under Extended Adaptive Attacks (CIFAR-100 + ResNet-18).

Penalty Weight (λ)	ASR (%)	True Detection AUC (%)
0.0	100.00	96.75
1.0	100.00	83.62
3.0	100.00	82.26
5.0	98.70	82.12
10.0	92.40	80.54
30.0	62.90	84.84
100.0	56.70	85.18
300.0	54.50	87.61
1000.0	52.30	87.01

**Table 16:** Surrogate-Detector Transfer Attack Evaluation on CIFAR-10 + ResNet-18.

Penalty Weight (λ)	ASR (%)	True Detection AUC (%)
0	100.00	91.08
0.0001	100.00	81.70
0.0003	100.00	63.09
0.001	100.00	31.53
0.002	100.00	16.00

**Table 17:** Summary of Typical Failure Cases of MSIS.

Failure Type	Typical Cause	Interpretation
Missed adversarial examples	Locally stable adversarial outputs	Sensitivity response resembles clean samples
False positives	Clean samples near class boundaries	Random perturbations induce large probability shifts
High-density task failures	Compressed inter-class decision regions	Clean and adversarial responses become less separable
Adaptive and transfer evasion cases	Increased evasion pressure or surrogate detector-score optimization	AUC drops to 80.54% under EoT attacks and to 16.00% under surrogate-transfer stress testing

**Table 18:** Comparison of Inference Latency and Access Requirements for Different Detection Methods.

Method	Required Access Permission	Inference Latency (ms/Sample)
MSP	Output probabilities only (Non-intrusive)	0.101
Energy Score	Final output layer only (Non-intrusive)	0.102
Feature Squeezing	Output probabilities only (Non-intrusive)	0.199
Mahalanobis	Intermediate layer features (Intrusive)	0.115
PASA	Model gradients (Intrusive)	47.344
**MSIS (Ours)**	**Output probabilities only (Non-intrusive)**	**4.076**

## Data Availability

The CIFAR-10 and CIFAR-100 datasets used in this study are publicly available. The experimental results generated during the current study are reported in the article. Additional implementation details are available from the corresponding author upon reasonable request.
